# Infrared Spectroscopy as a Promising Tool for Diagnosing and Typing Human Pathogenic Fungi

**DOI:** 10.1111/myc.70151

**Published:** 2026-01-16

**Authors:** Anthony G. J. Medeiros, Ayrton L. F. Nascimento, Luana Rossato, Daniel Assis Santos, Nalu Teixeira de Aguiar Peres, Reginaldo Goncalves de Lima Neto, Jacques F. Meis, Kássio M. G. Lima, Rafael Wesley Bastos

**Affiliations:** ^1^ Centro de Biociências Universidade Federal Do Rio Grande Do Norte Natal Brazil; ^2^ Laboratório de Química Biológica e Quimiometria, Instituto de Química Universidade Federal Do Rio Grande Do Norte Natal Brazil; ^3^ Laboratório de Pesquisa Em Ciências da Saúde Universidade Federal da Grande Dourados Dourados Brazil; ^4^ Laboratório de Micologia, Instituto de Ciências Biológicas Universidade Federal de Minas Gerais Belo Horizonte Brazil; ^5^ National Institute of Science and Technology in Human Pathogenic Fungi Ribeirão Preto Brazil; ^6^ Medicina Tropical Do Centro de Ciências Médicas da Universidade Federal de Pernambuco Recife Brazil; ^7^ Institute of Translational Research, Cologne Excellence Cluster on Cellular Stress Responses in Aging‐Associated Diseases (CECAD), excellence Center for Medical Mycology (ECMM) University of Cologne Cologne Germany; ^8^ Centre of Expertise in Mycology Radboudumc/CWZ Nijmegen Nijmegen the Netherlands

**Keywords:** Biotyper, fungal identification, infrared radiation, medical mycology

## Abstract

Fungal infections are increasingly recognised as a global health challenge, responsible for millions of cases annually and substantial mortality, especially in immunocompromised individuals. Yet, the diagnosis of these infections remains notoriously difficult, often delayed by slow culture‐based methods or hindered by the high cost and infrastructure demands of molecular diagnostics. In recent years, infrared (IR) spectroscopy has emerged as a promising alternative, offering rapid, cost‐effective and reagent‐free identification of human pathogenic fungi. This review provides an in‐depth examination of how IR‐based techniques, specifically, mid‐infrared (MIR) and near‐infrared (NIR) spectroscopy, are being applied in medical mycology. We explore the underlying chemical principles and highlight how recent advances in multivariate analysis and machine learning have enhanced their diagnostic accuracy. Studies have demonstrated the capacity of IR spectroscopy to accurately identify and type major fungal pathogens, while also providing insights into antifungal resistance profiles and outbreak tracking. While challenges remain, particularly regarding protocol standardisation and expansion of spectral databases, IR spectroscopy stands out as a valuable diagnostic strategy, especially in resource‐limited settings. By reducing diagnostic time and cost, and expanding accessibility, IR‐based methods have the potential to transform the clinical management of fungal infections, contributing to faster decision‐making and improved patient outcomes.

## 1 Introduction

Fungi are eukaryotic organisms that can exhibit two distinct morphologies: unicellular, known as yeasts, and multicellular forms, referred to as filamentous fungi or moulds. Both types of organisms are responsible for a wide range of diseases known as mycoses. The classification of these infections depends on the etiological agent, the host's immune conditions, and clinical manifestations [1, 2].

Based on these criteria, mycoses can be classified into four main categories: (i) superficial or mucocutaneous mycoses, which primarily affect the nails, skin and/or mucous membranes; (ii) subcutaneous mycoses, usually arising from traumatic injuries that enable fungal invasion of deeper skin layers; (iii) invasive or deep mycoses, which involve internal organs such as the lungs, central nervous system, genitourinary tract, and bloodstream, typically occurring in individuals with immunosuppression or other underlying conditions; and (iv) systemic mycoses that involve both cutaneous and internal organs, caused by a specific group of thermally dimorphic fungi that exhibit two distinct morphologies depending on temperature. Each of these categories is associated with specific human fungal pathogens [3, 4, 5].

Human fungal diseases are becoming increasingly prevalent, with new pathogenic species being continually identified, contributing to a growing number of cases. It is estimated that dermatomycoses affect approximately 25% of the global population, while invasive fungal infections impact around 300 million people annually, resulting in high mortality rates that account for up to 3.8 million deaths worldwide each year [6, 7]. Several factors contribute to this high mortality rate, including the limited availability of antifungal treatments, the emergence of drug resistance, the unavailability of antifungal agents in some countries, and the high cost of treatments where they are available. Many of these infections also disproportionately affect immunocompromised patients, further complicating their treatment [8].

While these factors are important, accurate diagnosis is essential before appropriate treatment and patient management can be implemented. Identifying the causative agent is crucial. However, it is estimated that the incidence of fungal infections and the associated mortality rates are significantly underreported, with diagnostic limitations representing the main bottleneck in the effective management of fungal diseases [9].

Several challenges impair diagnosis and the identification of fungal pathogens, such as the inherent time spent in culturing fungi, the need for highly trained laboratory staff, the high cost of automated, immunological and molecular diagnostic methods, and the lack of standardised identification techniques [10]. Ultimately, these barriers result in delayed or inaccessible diagnostic procedures, leading to improper or late treatment.

Classical methods for fungal identification rely on macroscopic and microscopic morphology, as well as on the physiological and biochemical properties. The observation of reproductive structures, such as spores and hyphae (both vegetative and reproductive), remains a fundamental step in the identification of moulds. Similarly, biochemical tests are commonly employed, particularly for the identification of yeasts. In addition, growth tests on selective and differential media are used not only to isolate fungi but also to characterise them based on their nutritional preferences and responses to different environmental conditions. However, these methods require highly trained personnel and are often time‐consuming [11, 12].

Fungal identification methods have made significant leaps in accuracy and speed with the advent of molecular biology. Ribosomal DNA sequencing analysis, especially the Internal Transcribed Spacer region (ITS), has been widely used to identify fungi [13]. The polymerase chain reaction (PCR) technique allows amplification of specific regions of the fungal genome for subsequent analysis [14]. Compared to the classical methods, these new techniques are more agile and have high specificity [15]. Next‐generation sequencing (NGS) has also emerged as a powerful tool, enabling simultaneous analysis of multiple samples and identification of complex fungal communities in natural environments [16, 17].

Recently, spectrometric techniques have gained prominence in fungal identification. Matrix‐assisted laser desorption/ionisation time‐of‐flight mass spectrometry (MALDI‐TOF MS) allows rapid and accurate identification of fungi based on mass spectra of specific proteins [18, 19, 20]. This approach has been widely used in clinical laboratories due to its speed and precision, and it has emerged as a reliable tool for fast identification and resistance detection [21, 22].

However, both molecular biology and mass spectrometry methodologies are costly; they rely on accurate databases and often are not available in regions with a high burden of fungal infections, such as low‐income countries, where accuracy and rapid fungal identification are crucial [23]. To improve this scenario, it is necessary to focus on developing accessible methods that require less specialised infrastructure, allowing for broader application, especially in countries with limited resources.

In this context, infrared‐based techniques emerged as a promising tool for characterising chemical processes and compounds, and they are now becoming increasingly useful for the identification of biological derivatives, particularly in microbiology [24]. Studies on the use of infrared‐based methods for identifying fungi and bacteria date back to the 1950s. However, thanks to advances in computing and emerging technologies, these techniques have become more accessible and efficient in recent years [25, 26, 27, 28].

The main goal of this review is to present and discuss infrared (IR) spectroscopy as a tool for diagnosing and identifying human pathogenic fungi. First, we describe the chemical and physical principles of IR spectroscopy and then discuss studies that present data on the use of infrared‐based techniques for the identification of fungi, as well as the potential of these techniques for use in clinical and research laboratories. Our literature review focuses on articles published from January 2000 to July 2025 that address infrared‐based methods and their application in the identification of medically important fungi.

## 2 Infrared Spectroscopy: Chemical and Physical Aspects

Infrared spectroscopy is a vibrational technique that can analyse biological systems, as complex molecules such as proteins, lipids, carbohydrates and nucleic acids exhibit distinct vibrational behaviours according to their structure and molecular conformation [29]. Molecules are made of atoms connected by chemical bonds, which are not static; possessing rotational and vibrational energy [30, 31]. When IR radiation (IR) is absorbed, it excites these bonds, causing changes in the vibrational state of the molecule [32]. The energy of this radiation is commonly described using wavelength (*λ*) or wavenumber (cm^−1^). Wavenumber is inversely proportional to wavelength and directly proportional to the energy of the vibration [33].

**FIGURE 1:**
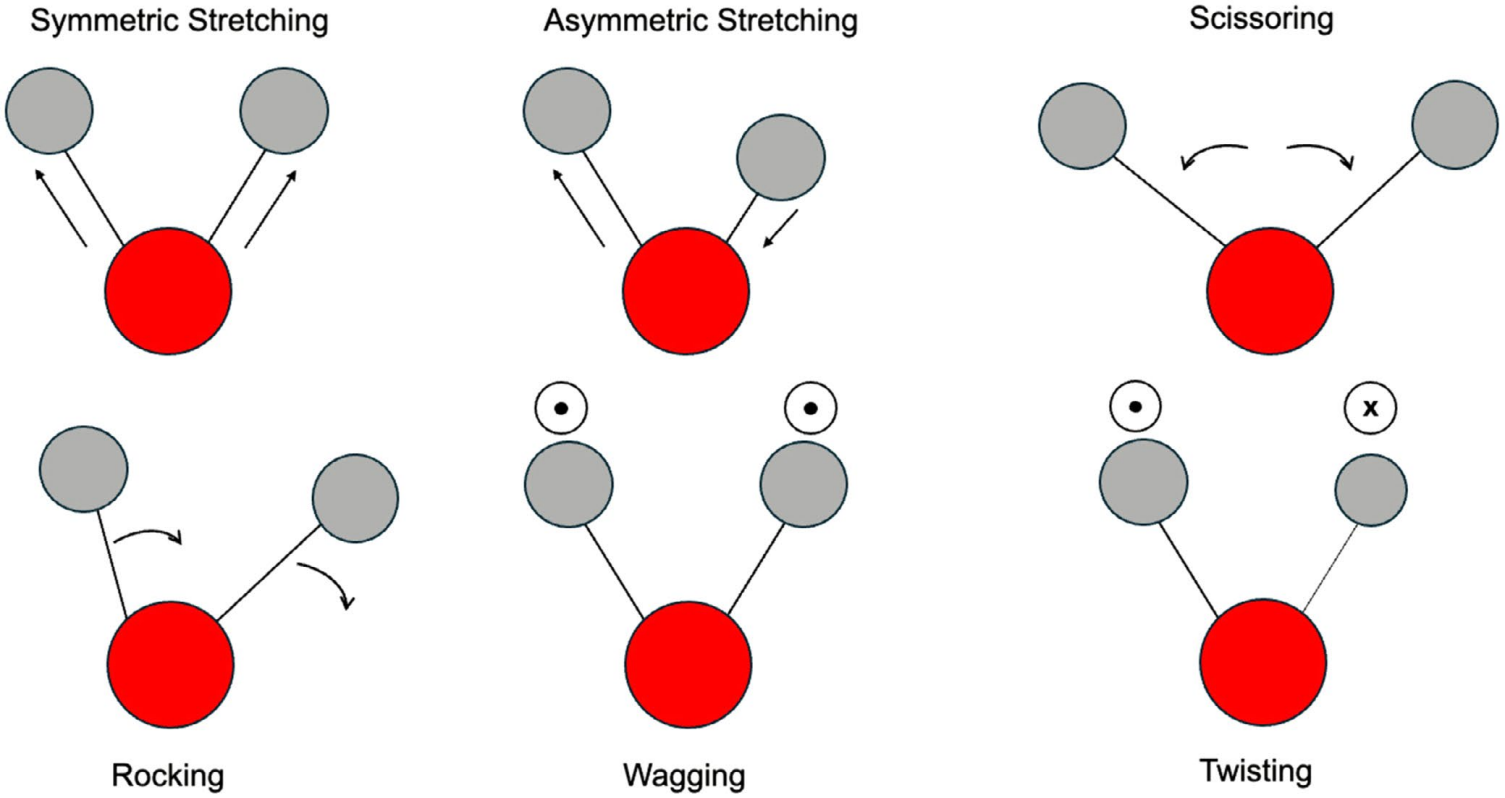
The different vibrational modes for a molecule using a CH2 group in an organic compound as example. The red balls represent a Carbon atom while the grey ones represent Hydrogen atoms. The circle with a ‘•’ in the centre indicates a movement towards the direction of the reader while the circle with a ‘*x*’ in the centre indicates a change in direction to behind the plane of reading. (adapted from Giustiniani [34]). Bending vibrations are either in‐plane or out‐of‐plane. In‐plane bending consists of scissoring (two atoms move towards and away from each other, similar to the motion of a pair of scissors) and rocking (two atoms move in the same direction, maintaining the bond angle but changing the overall spatial orientation). Out‐of‐plane bending consists of wagging (atoms move up and down together, resembling the motion of a wagging tail) and twisting (One atom moves up while another moves down, as if the group of atoms were being twisted about the bond axis).

After absorbing IR energy, molecules can vibrate in two principal modes: stretching, where the interatomic distance changes, and bending (or deformation), where the bond angle changes. Stretching can be symmetric or asymmetric, while bending vibrations include in‐plane (scissoring and rocking) and out‐of‐plane (wagging and twisting) motions (Figure 1) [31, 34].

**FIGURE 2:**
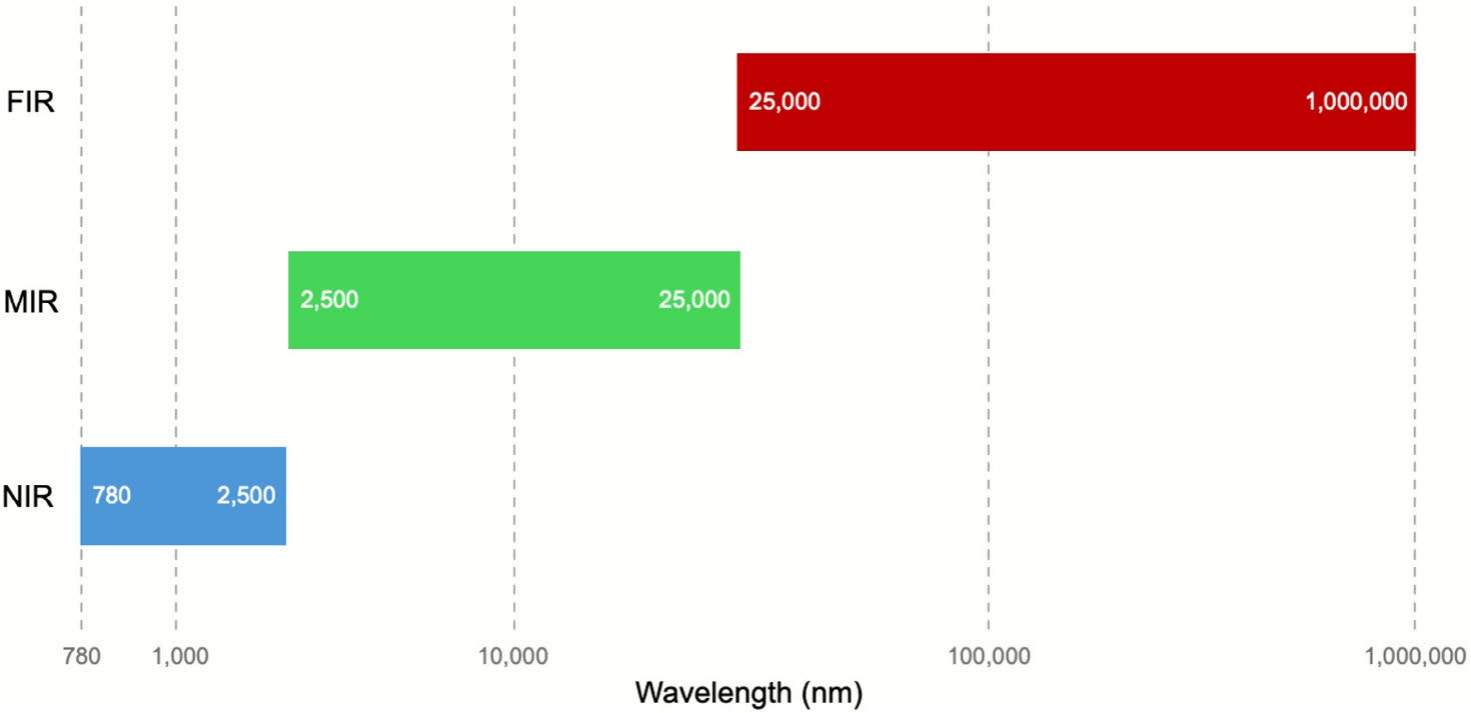
Spectral range for I radiation separated by wavelength regions. The blue, green and red bars depict the near‐infrared (NIR—from 780 to 2500 nm), mid‐infrared (MIR—from 2500 to 25,000 nm) and far‐infrared (FIR—from 25,000 to 1,000,000 nm) regions, respectively.

The IR has three regions ranging from 780 nm to 25,000 nm (Figure 2). The near‐infrared (NIR) region covers the region from approximately 780 to 2500 nm (12,800–4000 cm^−1^) (Figure 2), associated with weaker overtones and combination bands of fundamental vibrations, particularly from N‐H, O‐H and C‐H bonds [30, 31]. The mid‐infrared (MIR) portion comprises the region between 2500 and 25,000 nm (4000–400 cm^−1^) (Figure 2), corresponding to the fundamental vibrational transitions of most functional groups (e.g., carboxyl, amine and amide). Since it provides a unique ‘molecular fingerprint’ for different compounds, it is the most widely used region in IR spectroscopy [32, 35]. The far‐infrared (FIR) segment is located at the lowest energy part of the IR spectrum (25,000–1,000,000 nm or 400–10 cm^−1^) (Figure 1). This region is associated with the rotational energy of molecules and low‐energy skeletal vibrations [31]. Although FIR has some applications, such as providing information on protein structures, it is usually complemented with other techniques such as nuclear magnetic resonance or fluorescence‐based analyses [36]. The use of FIR is less frequent compared to the other two already mentioned IR techniques, so this review will mainly focus on MIR and NIR applications.

### 2.1 The IR Spectrum

**FIGURE 3:**
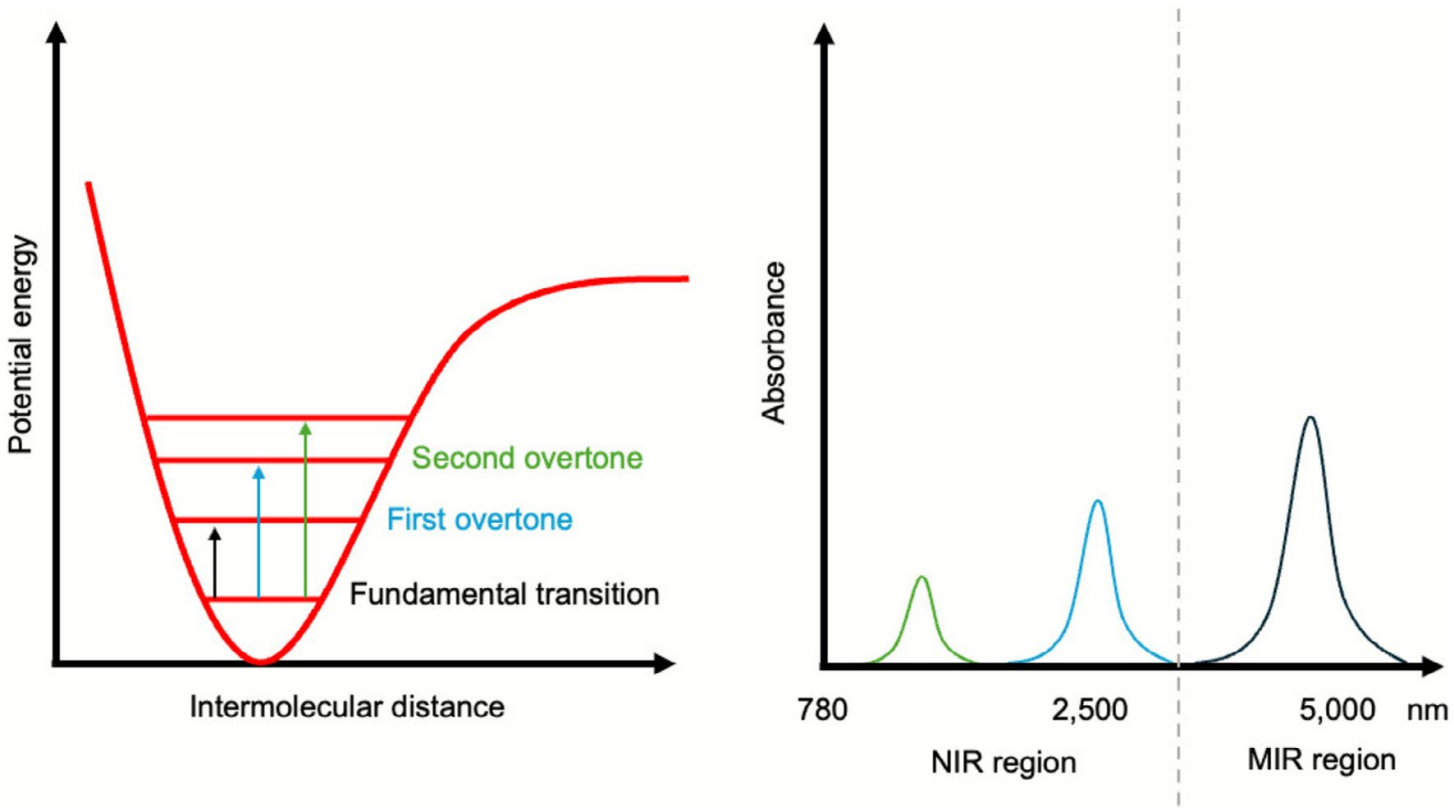
Schematic representation of molecular vibrational transitions based on the anharmonic oscillator model (left), illustrating fundamental transitions and overtone absorptions (first and second) that occur upon infrared (IR) light interaction with a molecular bond. On the right, the corresponding absorbance spectrum shows typical absorption bands, highlighting the near‐infrared (NIR) region (780 to 2500 nm), where overtones and combination bands dominate, and the mid‐infrared (MIR) region (2500–5000 nm), characterised by fundamental vibrational transitions.

When light from a spectrophotometer interacts with a sample, it is absorbed if its energy matches the vibrational frequency of a molecular bond [31]. This can be initially understood using the harmonic oscillator model, where two atoms are like masses attached by a spring. However, real molecular vibrations are anharmonic, meaning they deviate from this ideal model. This anharmonicity allows for transitions to higher energy states, which are typically forbidden in the harmonic model. These transitions generate overtones, which can be thought of as harmonics of a fundamental musical note—they occur at approximately integer multiples of the fundamental frequency but with much lower intensity. These weaker overtones and their combinations constitute the NIR spectrum and are particularly important for the analysis of organic materials, as they are associated with bonds such as C‐H, O‐H and N‐H, while the stronger, fundamental transitions dominate the MIR spectrum [31, 37, 38]. Figure 3 shows the relation between the molecular absorptions and the IR spectrum generated.

Fourier Transform Infrared (FTIR) Spectroscopy uses an interferometer to collect the radiation, which is then processed using a mathematical method called Fourier Transform to generate the final spectrum. FTIR captures the entire spectrum simultaneously, not requiring the sample to be exposed to different frequencies one at a time [33, 39]. For microbial typing, FTIR spectroscopy can bring information about cell composition, serving as molecular fingerprints. Each microorganism exposes a unique spectrum due to variations in cell wall and membrane, in addition to intracellular components. By comparing the spectral signatures from different strains, it is possible to differentiate and classify them [40].

### 2.2 Multivariate Analysis

An IR spectrum contains thousands of data points (absorbance at each wavenumber), making visual interpretation nearly impossible, especially for complex biological samples like fungi. Therefore, the use of multivariate statistical methods, or chemometrics, is essential to extract meaningful patterns from this high‐dimensionality data and has been growing in recent years, whether for classification or calibration [41, 42, 43, 44, 45].

There are two classes of multivariate analysis techniques for pattern recognition: unsupervised and supervised methods. The former aims to detect similarities and differences within a dataset composed, for example, of spectra from different classes without prior information about the class to which they belong. Principal component analysis (PCA) is the most popular unsupervised method, along with hierarchical cluster analysis (HCA) and *K*‐means (Table 1) [46]. On the other hand, in supervised methods, different classes are previously informed. They are based on two successive steps: first, samples whose class is known are used to build a model with suitable parameters that optimise the discrimination between data from different classes; then, unknown samples are assigned to an appropriate class using the parameters optimised during the first stage. Linear discriminant analysis (LDA) and quadratic discriminant analysis (QDA) are effective supervised approaches (Table 1) [47, 48] Other very often used techniques are variations of regression models modified for classification, such as partial least squares discriminant analysis (PLS‐DA) and orthogonal partial least squares discriminant analysis (OPLS‐DA), the latter being an evolution of the PLS‐DA model (Table 1) [48, 49].

**TABLE 1:** Summary of multivariate analysis for dimensionality reduction, classification and variable selection.

Method	Purpose	How it works	Key characteristics	References
HCA (hierarchical cluster analysis)	To recognise patterns, trends and classes formation, facilitating the relationship interpretation among samples by a visual representation of the data.	It calculates the distance from one sample to another in a multivariate space. Samples similar to each other are closer together.	Results can be easily interpreted by a dendrogram plot (a tree‐like diagram). The results can be affected by the type of distance calculation.	[46]
*K*‐means	Similar to HCA, but it uses an iterative technique to partition the data set.	Seeks to minimise the distance of the samples of a data set with virtual central points for each cluster.	Based on sample‐centre distances, assigning each i‐th element to the nearest cluster. It is more robust than HCA but it requires an estimative of the number of clusters by the user.	[46]
PCA (principal component analysis)	Reduce the dimensionality of large datasets while retaining maximum variance.	Transforms original variables into orthogonal Principal Components (PCs). The matrix X is decomposed into scores (T), loadings (P) and residuals (E).	New variables are uncorrelated. PCs are ordered by variance. Common for exploratory analyses or preprocessing.	[47]
LDA (linear discriminant analysis)	Classify samples into predefined classes by maximising class separability.	Uses Mahalanobis distance and pooled covariance to compute linear discriminants, thus assigning each sample to its class.	Assumes equal covariance across classes. Limited when number of variables > samples. Often preceded by variable reduction algorithms such as PCA and others.	[29]
QDA (quadratic discriminant analysis)	Similarly to LDA, However, QDA forms a separate variance model for each class.	Also based on a Mahalanobis distance. By using different covariance matrix for each class, forms a quadratic discrimination function.	Outperforms LDA when classes exhibit a large difference in the number of samples, but it suffers with small‐sized datasets. Like LDA, is highly affected by ill‐conditioned data and also needs variable reduction algorithms.	[48]
PLS‐DA (partial least squares discriminant analysis)	Modification of the PLS regression model used to feature extraction and sample classification. Different of PCA, PLS‐DA tries to find a linear relationship between the variables and the response matrix (classes).	It reduces the original spectral variables to a small number of Latent Variables (LVs), where then a linear discriminant classifier is used for classifying the groups.	Usually performs better than PCA followed by LDA. However, PLS‐DA is a binary classifier, making the comparison of more than two classes is difficult.	[48]
OPLS‐DA (orthogonal partial least squares discriminant analysis)	An evolution of PLS‐DA that filters the real contribution of the variables to the model, using predictive and orthogonal variances.	The predictive part of the variance is responsible for the variation of the classes, while the orthogonal part represents fluctuations not directly responsible for class variation. After this step, LVs are computed as in the PLS‐DA model.	By removing orthogonal variation, simplifies interpretation, result in simple, robust and focused models. As PLS‐DA, is the comparison among more than two classes is difficult.	[49]
SPA (successive projections algorithm)	Select variables with minimal collinearity to improve model accuracy by removing redundant variables.	Uses projection operations to form variable chains, evaluates subsets, and removes uninformative variables via backward elimination.	Effective for collinear data. Involves combinatorial optimization. Three‐step process: projection, evaluation, elimination.	[50, 51]
GA (genetic algorithm)	Heuristic optimization technique for selecting the most relevant variables.	Mimics natural selection: encodes variables as chromosomes, evaluates them via a fitness function, and evolves the population through crossover and mutation until a stopping criterion is met.	Probabilistic, non‐local search. Stochastic in nature. Requires user‐defined parameters like population size and max generations. Five‐step iterative process.	[52, 53]

## 3 Potentials and Limitations of Infrared Spectroscopy in Mycology

**FIGURE 4:**
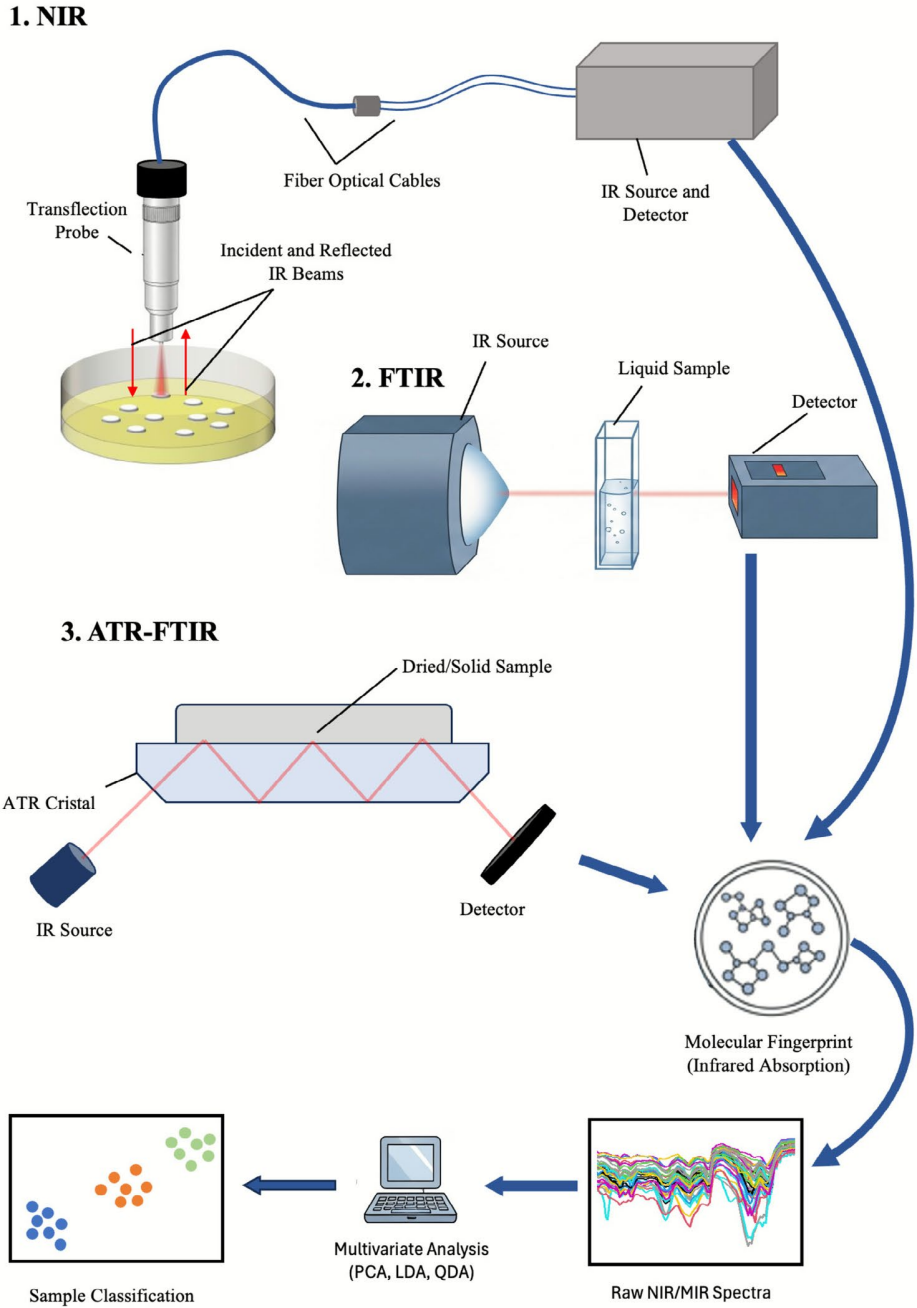
Analytical workflow based on vibrational spectroscopy for sample characterisation and classification. Three complementary approaches are illustrated: (1) near‐infrared spectroscopy (NIR), using a transflection probe coupled to fibre optic cables for direct spectral acquisition; (2) Fourier‐transform infrared spectroscopy (FTIR), applied to liquid or solid samples, where IR radiation interacts with the matrix and is subsequently detected; and (3) attenuated total reflectance FTIR (ATR‐FTIR), in which dried or solid samples are analysed in direct contact with the ATR crystal, enabling rapid measurements without extensive sample preparation. The acquired signals provide a molecular fingerprint (infrared absorption), which is processed into raw NIR/MIR spectra and further subjected to multivariate analysis (PCA, LDA and QDA) for pattern extraction and dimensionality reduction. The resulting data allow sample classification, discriminating groups or experimental conditions of interest.

The use of different IR spectroscopy techniques combined with data analysis enables fungal classification, which can be divided into two main parts: IR spectra acquisition and multivariate analysis [24, 25, 54, 55, 56]. Figure 4 illustrates, in a simplified manner, the use of different IR spectroscopy techniques. IR techniques can be employed in three main modes: transmission, transflection and by attenuated total reflection (ATR) [57]. In transflection, common in NIR, but also with new developments in MIR, IR radiation is sent through a probe to the sample by optical cables and reflected beams of light travel back the same probe, being collected [58, 59]. Depending on the instrument configuration, the source and detector may be positioned together in the same housing or separated. In transmission FTIR, an infrared source emits a beam that passes through the sample before reaching the detector, providing direct information on the absorption characteristics of the analyte [57, 58]. ATR‐FTIR is applied to dried or solid samples placed in contact with an ATR crystal. In this configuration, the IR beam enters the crystal and undergoes multiple internal reflections, generating evanescent waves at each reflection point. These waves penetrate a few micrometres into the sample, and the attenuated signal is subsequently detected (Figure 4) [60].

The interaction between IR radiation and molecular bonds produces unique absorption patterns that constitute a molecular fingerprint for each sample [24]. The resulting spectra, represented as absorbance or transmittance against wavenumber or wavelength, are complex datasets that require computational processing (Figure 4). Multivariate statistical methods are employed to reduce data dimensionality and identify discriminative features (Figure 4) [61, 62]. This workflow transforms raw spectral data into meaningful classifications, enabling the discrimination of fungal groups and experimental conditions.

IR spectroscopy is mostly reagent‐free, reducing the need for chemical consumables for most techniques and making the process more cost‐effective using cheap reagents like ethanol, thus environmentally friendly. The robustness of ATR‐FTIR provides high reproducibility and accuracy for genus‐ and species‐level identification [63] and can even detect subtle biochemical alterations, such as those during phenotypic transitions from yeast to hyphae [64]. However, the MIR approach has some limitations. A significant challenge is the potential for overlapping spectral features between closely related fungal species, which can complicate differentiation. Additionally, the spectra can be sensitive to sample preparation methods and environmental conditions, requiring standardised protocols to ensure consistent results [65].

For a long time, NIR spectroscopy was often overlooked in favour of FTIR due to its more complex spectra, which consist of weaker overtones and combination bands that are harder to interpret. This has limited the use of NIR in certain areas of biological research. A further disadvantage is its higher sensitivity to environmental variations like temperature and humidity, which can introduce noise and interfere with spectral data [66, 67]. Despite these challenges, advancements in chemometrics have unlocked NIR's potential. A key advantage of NIR radiation is its ability to allow for deeper sample penetration, enabling the analysis of thicker materials or bulk samples with little to no surface preparation [37, 38]. This ability has led to its wide employment in food, pharmaceutical, and agricultural analysis. Moreover, NIR is often more cost‐effective, as its instrumentation is more easily adapted for portable devices [32, 68].

Ultimately, although both techniques present advantages and disadvantages, their use in routine diagnostics is expected to grow. The primary challenge for both is that many biomolecules contribute to the entire signal, leading to a large amount of complex data that can lack immediate specificity. However, calibration models, advanced preprocessing, and multivariate analysis have proven to be effective in overcoming these limitations, making infrared spectroscopy a powerful tool for modern microbiology [29, 67, 69]. Table 2 compares the advantages and disadvantages of NIR and MIR spectroscopy.

**TABLE 2:** Main characteristics, advantages and limitations of NIR and MIR spectroscopy.

Feature/Aspect	NIR	MIR (FTIR, ATR‐FTIR)	References
Spectral features	Weaker overtones and combination bands; more difficult to interpret	Stronger fundamental absorptions; high structural selectivity	[30, 31, 32, 35]
Penetration depth	Deeper penetration; suitable for bulk sample analysis	Shallow penetration (a few micrometres with ATR)	[37, 38]
Sample preparation	Often minimal or none; can analyse intact samples	Also minimal with ATR; no complex preparation needed	[63]
Speed of analysis	Very fast; results can be analysed in minutes	Very fast; results can be analysed in 1–2 min	[63]
Specificity for biomarkers	Lower specificity; overlapping signals from many biomolecules	Higher specificity; better at distinguishing functional groups	[32, 35, 66, 67]
Sensitivity to environmental conditions	More affected by temperature, humidity, and other external factors	Less affected, though standardisation is still important	[65, 66, 67]
Instrument cost and portability	More cost‐effective; uses cheaper light sources; portable devices are easier to build	Generally, more expensive; less portable	[32, 68]
Use of reagents	Reagent‐free	Reagent‐free	[63]
Data complexity	High; requires chemometric models and preprocessing for meaningful interpretation	Also requires processing, but spectra are more directly interpretable	[29, 67, 69]
Applications	Widely used in food, agriculture and pharmaceutical analysis for bulk non‐destructive testing, recently fungal species classification	Common in many areas, from food, biology, clinical diagnoses, proteomics, environmental and forensic applications	[25, 35, 36, 55, 56, 70, 71, 72]
Limitations	Harder to identify specific molecules; sensitive to external variations.	Possible spectral overlap between spectral features; requires standardised protocols.	[65, 66, 67]
Recent advances	Chemometrics has improved classification and quantification capabilities.	ATR‐FTIR has shown high accuracy and reproducibility in genus and species identification.	[48, 69]

### 3.1 Applications of Infrared Spectroscopy for the Identification of Human Pathogenic Fungi

#### 3.1.1 *Candida* spp.

*Candida* is a genus of yeast‐like fungi, comprising species that may undergo dimorphic transitions to pseudohyphae or hyphae. These organisms are responsible for a wide range of mycoses collectively known as candidiasis, which vary from superficial or mucocutaneous infections, affecting the skin, oral cavity, vagina and gastrointestinal tract, to severe invasive infections associated with high morbidity and mortality [67, 73].

Among the numerous species described, *Candida albicans* , *C. tropicalis* , *C. glabrata* (Synonym *Nakaseomyces glabrata*), *C. parapsilosis* , and *C. krusei* (Synonym *Pichia kudriavzevii*) are the most frequently associated with clinical cases [74, 75, 76]. *C. albicans* continues to be the predominant species in most settings; however, emerging species like *Candida auris* (Synonym *Candidozyma auris*) [77] have become a global concern due to multidrug‐resistant profiles, high transmissibility and mortality rates that can reach 50%, contributing to numerous hospital outbreaks [78, 79].

IR spectroscopy has been applied to the identification of *Candida* species and their metabolic profiles, with over three decades of scientific exploration supporting its validity. This can contribute to the identification of clinical *Candida* isolates by presenting extremely specific spectral fingerprints that represent the molecular structure of these microbes [24].

Several studies have demonstrated the efficacy of FTIR spectroscopy in the accurate identification and classification of *Candida* spp. (Table 3). Potocki et al. [80] utilised ATR‐FTIR to cluster clinical isolates of *C. albicans* , *C. tropicalis* and *C. glabrata* based on their spectral patterns, which correlated with biochemical and genotypic characteristics. This approach was further expanded by Lam et al. [54] who developed a multicenter study involving 573 clinical yeast isolates to build a comprehensive spectral database. Their methodology achieved an impressive overall correct identification rate exceeding 98%, reaching 100% for the most prevalent species, such as *C. albicans* , *C. parapsilosis* and *C. tropicalis* . Nevertheless, they identified limitations related to underrepresented groups, as 9 isolates, particularly non‐ *C. parapsilosis* members of the *C. parapsilosis* complex, could not be correctly classified, reinforcing the necessity of comprehensive spectral libraries for optimal performance.

**TABLE 3:** Summary table of studied fungi and their IR‐approaches validated methods and current status.

Studied fungi	Infrared spectroscopy	Current development/applicability status	References
*Candida* spp.	NIR, ATR‐FTIR	Proof‐of‐concept	[24, 48, 80, 81, 82, 83, 84]
	IR Biotyper	Commercially available solutions	[85, 86]
*Cryptococcus* spp.	ATR‐FTIR	Proof‐of‐concept	[54, 87]
*Rhodotorula mucilaginosa*	ATR‐FTIR	Proof‐of‐concept	[54]
*Aspergillus* spp.	FTIR	Proof‐of‐concept	[88]
Dermatophytes‐causing genera (*Trichophyton, Epidermophyton, Nannyzzia*, *Microsporum*)	ATR‐FTIR	Proof‐of‐concept	[89]
CBM‐causing genera (*Fonsecaea* spp., *Cladophialophora* spp., *Phialophora* spp., *Exophiala* spp., *Rhinocladiella* spp.)	ATR‐FTIR	Proof‐of‐concept	[90]
*Paracoccidioides* spp.	FTIR	Proof‐of‐concept	[91, 92]

Vatanshenassan et al. [93] was among the first studies to demonstrate that IR spectroscopy could serve as a reliable methodology for identifying and clustering emerging yeasts such as *C. auris* , by comparing the performance of FTIR with other well‐established identification techniques, including microsatellite typing, ITS sequencing, AFLP fingerprinting and MALDI‐TOF mass spectrometry. Both methodologies present advantages and disadvantages, and each one grouped the isolates in a distinct manner. However, FTIR spectroscopy stood out as one of the approaches with clustering performance comparable to microsatellite typing for *C. auris* isolates.

Similarly, Franconi et al. [81] conducted a comparative analysis between ATR‐FTIR spectroscopy, processed with the I‐dOne software, and the well‐established MALDI‐TOF MS technique. By analysing spectra from 284 *Candida* isolates ( *C. albicans* *n* = 139, *C. parapsilosis* complex *n* = 66, *C. glabrata* *n* = 50, *C. tropicalis* *n* = 20 and *C. krusei* *n* = 9), collected from single colonies, they obtained species‐level identification in agreement with MALDI‐TOF MS in 95.8% (272/284) of cases. This underscores the robustness of ATR‐FTIR as a reliable, cost‐effective, reagent‐free and partially non‐destructive alternative for species identification in clinical microbiology.

FTIR spectroscopy has also shown promise in discriminating antifungal resistance profiles, in a manner similar for MALDI‐TOF MS, which was previously demonstrated by Vatanshenassan et al. [93]. For instance, De Carolis et al. [82] used FTIR to type fluconazole‐resistant *C. parapsilosis* isolates, successfully clustering them according to specific resistance‐associated mutations, particularly by analysing the carbohydrate spectral region (1300–800 cm^−1^). Expanding on this application to other *Candida* species, Dróżdż et al. [83] employed ATR‐FTIR to identify resistance‐related spectral signatures in *C. albicans* , *C. dubliniensis*, and *C. glabrata* . By categorising strains as sensitive or resistant according to EUCAST MIC assays, they observed significant differences in the intensity of specific absorbance bands. For example, fluconazole‐resistant *C. albicans* isolates exhibited increased absorbance at 1716, 1515, 1125 and 809 cm^−1^, along with a decrease at 1368 cm^−1^. These spectral shifts appeared to be species‐ and drug‐specific, reinforcing the potential of FTIR spectroscopy as a tool for resistance detection. Despite limitations in sample size, the findings highlight the capacity of FTIR to complement conventional phenotypic and genotypic methods, underscoring the need for more comprehensive spectral databases to enhance diagnostic accuracy.

Beyond the selection of analytical techniques, sample preparation represents a critical factor that can influence the quality and interpretation of spectral data. In this context, Pebotuwa et al. [94] systematically investigated the impact of different preparation methods, namely untreated, washed and formalin‐fixed colonies, on ATR‐FTIR spectra. By applying PCA and *K*‐means clustering (KMC), the authors demonstrated that while sample preparation can alter certain spectral features, such as the enhancement of polysaccharide bands in fixed samples, it does not compromise the overall ability to achieve accurate species‐level discrimination. These results underscore the importance of adopting standardised preparation protocols, adapted to the specific goals of each analytical application, to ensure reproducibility and consistency in spectral analyses.

The translation of these findings into clinical practice has been facilitated by the development of commercial systems such as the IR Biotyper (Bruker Daltonics GmbH & Co. KG), which integrates ATR‐FTIR spectroscopy with user‐friendly software for microbial typing. The system operates by directly applying a microbial colony onto a silicon crystal, acquiring mid‐infrared spectra (typically within the 4000–400 cm^−1^ range), and analysing the biochemical fingerprints, —primarily of lipids, proteins, and carbohydrates—using multivariate statistical tools such as PCA and LDA. This enables rapid clustering of isolates based on spectral similarity, with minimal sample preparation without the need for molecular reagents.

IR Biotyper was successfully applied in a study by Curtoni et al. [85] to investigate an outbreak of *C. auris* in an ICU setting, demonstrating high concordance with whole‐genome sequencing (WGS)‐based genotyping through PCA and LDA analyses. This established FTIR as a practical and efficient alternative for rapid outbreak surveillance. In a similar context, De Melo et al. [86] evaluated the performance of the IR Biotyper in clade classification of *C. auris* isolates from Brazilian hospital outbreaks. Analysing 69 isolates, predominantly from the state of Pernambuco, the authors demonstrated accurate clustering into the respective clades (I–IV), with the majority of Brazilian isolates belonging to clade IV. This approach provided timely epidemiological insights that would otherwise require labour‐intensive and costly molecular methods such as MLST or WGS.

Building upon these advances in FTIR‐based classification, recent studies have explored ways to further enhance the discriminatory power by integrating machine learning algorithms. In this regard, Magrì et al. [84] developed the Clade‐Finder method, which combines FTIR spectroscopy with artificial neural networks (ANN) to improve clade‐level discrimination. Analysing 63 *C. auris* isolates representing clades I–IV (with clade V considered outliers due to limited sample numbers), their model achieved correct clade classification rates of up to 96% in validation datasets, underscoring the added value of AI‐enhanced FTIR approaches for epidemiological surveillance.

Expanding the scope beyond FTIR, Nascimento et al. [24] demonstrated the efficacy of NIR spectroscopy combined with multivariate analyses, SPA, GA, PCA and LDA, for distinguishing *C. auris* from *C. haemulonii sensu stricto*, two phylogenetically close yet clinically divergent species. Their approach achieved 100% sensitivity and specificity, reinforcing the utility of NIR as a complementary low‐cost tool for species identification, especially in the context of infection control.

It is important to note that while Nascimento et al. [24] focused on isolate‐level discrimination with a limited number of lineages, other studies incorporated more extensive datasets and applied broader analytical approaches, including clustering by resistance phenotype and epidemiological clade assignment. This demonstrates the versatility of IR‐based techniques in addressing multiple diagnostic challenges associated with *Candida* spp.

Collectively, these findings support the growing role of IR spectroscopy, both FTIR and NIR, as promising diagnostic platforms capable of complementing conventional methods, offering faster, less resource‐intensive, and accurate alternatives for species identification, antifungal resistance detection, and epidemiological surveillance, particularly for emerging multidrug‐resistant pathogens like *C. auris* .

#### 3.1.2 Application of Infrared Spectroscopy to Identify Other Clinically Relevant Yeasts

Other yeasts are also associated with infectious processes, some of which may be part of our normal microbiota and transit to a pathogenic process due to different conditions, while others are exclusively pathogenic acquired from the environment or animals [95].

The yeast‐like fungi *Cryptococcus neoformans* and *C. gattii* are commonly found in soil and bird droppings, where they can spread and infect susceptible humans. Infections caused by *Cryptococcus* species primarily affect the lungs, leading to pneumonia, from which the fungi may disseminate through the bloodstream to other organs. These fungi have a particular affinity for the central nervous system (CNS), resulting in a serious condition known as neuro‐cryptococcosis. While individuals living with HIV/AIDS are the most commonly affected by *Cryptococcus* species, *C. gattii* can also infect people without any underlying immunodeficiency [6].

As *C. neoformans* and *C. gattii* are mostly linked to opportunistic infections in immunocompromised patients, fast and precise identification is essential for improving the patient's prognosis if these yeasts are involved. Despite the genetic diversity of *Cryptococcus* spp., they have demonstrated capacity to frustrate traditional identification methods due to hybrids and cryptic species [87, 96, 97].

A pioneering study by Costa et al. [87] showed that ATR‐FTIR could effectively discriminate between *C. neoformans* and *C. gattii* isolates (Table 3). Interestingly, they found that the choice of the multivariate classification model was species‐dependent: models using PCA, SPA or GA associated with LDA performed best for *C. gattii* (achieving 100% accuracy), while models using QDA, especially SPA‐QDA and GA‐QDA, were necessary to achieve high sensitivity and specificity for *C. neoformans* .

Further supporting these findings, the multicenter study by Lam et al. [54], previously discussed for *Candida* identification, also demonstrated the successful application of ATR‐FTIR in identifying non‐*Candida* yeasts, such as *C. neoformans* and *Rhodotorula mucilaginosa* , with 100% accuracy, though based on a limited number of isolates.

It is important to highlight that recent phylogenetic analyses have proposed a significant taxonomic revision of the *Cryptococcus* genus. Specifically, *C. neoformans* and *C. gattii* have been reclassified into seven distinct species, based on robust genetic and phenotypic differences [98]. This updated classification reflects a more complex evolutionary landscape and introduces new challenges for clinical diagnostics, as conventional identification methods may not adequately resolve these newly proposed species [99]. While infrared spectroscopy has demonstrated the ability to capture subtle biochemical differences among fungal pathogens, it is important to note that its applicability for differentiating the newly classified *Cryptococcus* species has not yet been systematically evaluated in the literature, representing a potential avenue for future research.

#### 3.1.3 Application of Infrared to Identify Filamentous and Dimorphic Fungi

Recent developments in IR spectroscopy have greatly expanded the diagnostic potential for *Aspergillus* spp. (Table 3). The mould genus *Aspergillus* is prevalent in the environment and can lead to several diseases collectively referred to as aspergillosis. *Aspergillus fumigatus* is the most common causative agent, accounting for approximately 90% of cases; however, other species, such as *A. flavus* , *A. niger* , *A. nidulans* and *A. latus* , can also be responsible [75, 100]. The manifestation of aspergillosis varies depending on immune status and lung architecture, with the most common forms being invasive pulmonary aspergillosis, allergic bronchopulmonary aspergillosis, and chronic pulmonary aspergillosis. Invasive aspergillosis poses a significant threat, with a mortality rate that frequently approaches 50%, particularly for patients undergoing chemotherapy, those receiving transplants, and individuals with neutropenia [101, 102].

A significant step towards clinical application was taken by Elkadi et al. [88], who developed an IR‐based method to identify *Aspergillus* spp. directly from human blood plasma. They created sophisticated training sets to simulate real clinical scenarios, including plasma containing other common pathogens or therapeutic drugs. By using machine learning models as Partial Least Squares Discrimination Analysis (PLS‐DA), they were able to identify *Aspergillus* with high accuracy (up to 91.1%), even in the presence of these confounding factors, opening the possibility of diagnosing invasive aspergillosis and other fungal bloodstream infections much more rapidly.

Other fungal diseases, such as superficial mycoses and deep mycoses (subcutaneous and systemic), are caused by filamentous fungi that can also be identified with IR spectroscopy (Table 3). Onychomycosis is a superficial fungal infection of nails primarily caused by dermatophytes (*Trichophyton*, *Epidermophyton*, *Nannizzia*, *Microsporum*, and others), non‐dermatophytes, and yeasts [103]. The classical laboratory diagnosis relies on assessing the macromorphology of fungal colonies and the micromorphology of cells, requiring the expertise of trained professionals for a subjective analysis [104, 105, 106].

In a pioneering approach to overcome the diagnostic challenges associated with onychomycosis, ATR‐FTIR spectroscopy was employed for the direct analysis of nail clippings to identify fungal nail infections (Table 3) [89]. The authors developed a spectral library covering the most common etiological agents, including dermatophytes, non‐dermatophyte moulds and yeasts. Using PCA and HCA, the method successfully differentiated infected nails from healthy controls in both ex vivo and in vivo models and accurately classified the type of causative agent (dermatophyte, non‐dermatophyte or yeast), achieving accuracy rates above 96%. Despite these promising results, the application of IR spectroscopy for the identification of medically important filamentous fungi, particularly non‐dermatophyte moulds, remains relatively underexplored, highlighting a current knowledge gap and opening opportunities for future research.

Neglected tropical mycoses (NTMs), such as chromoblastomycosis (CBM), paracoccidioidomycosis (PCM) and sporotrichosis, affect millions in underdeveloped nations where diagnostic resources are scarce [107]. In this context, IR spectroscopy may offer a promising solution to bridge this diagnostic gap.

Chronic fungal infections of the skin and subcutaneous tissues, such as CBM, are caused by a diverse group of fungi belonging to at least seven different genera, including *Fonsecaea* spp., *Cladophialophora* spp., *Phialophora* spp., *Exophiala* spp. and *Rhinocladiella* spp. [108].

The accurate identification of these etiological agents remains a significant clinical and laboratory challenge, primarily due to their overlapping morphological features and the need for laborious, time‐consuming molecular techniques for species‐level discrimination. To overcome these limitations and provide a faster and more accessible alternative, Heidrich et al. [90] developed an ATR‐FTIR‐based method for the identification of the main CBM‐causing genera. While standard PCA was insufficient for reliable classification, the application of the supervised OPLS‐DA approach yielded models with 100% sensitivity, specificity and accuracy at the genus level.

For PCM, a systemic mycosis endemic to Latin America [109], two recent studies have demonstrated the potential for serum‐based diagnosis (Table 3). De Brito et al. [91] used FTIR and machine learning algorithms (Support Vector Machine—SVM) to analyse serum from PCM patients, identifying significant biochemical alterations and achieving 91.67% accuracy in discriminating them from healthy controls. Pushing this further, Koehler et al. [92] used a larger set of retrospective serum samples, including controls with other fungal infections, to test their ATR‐FTIR based method. Their approach, combining univariate analysis with LDA, achieved accuracies of 99%–100% in classifying PCM‐positive samples, showcasing the technique's high potential as a non‐invasive diagnostic tool.

Collectively, these studies suggest that IR spectroscopy, especially ATR‐FTIR, offers exciting potential as a rapid and accessible diagnostic tool for NTMs like CBM and PCM. The high accuracy rates reported, sometimes reaching 100%, are encouraging, particularly in the context of diseases where diagnostic delays remain a major barrier to proper treatment. Yet, a closer look at these studies reveals important gaps that should not be overlooked. Many investigations are based on small, well‐controlled sample sets, which may not fully capture the complexity of real‐world clinical cases seen in endemic regions.

For CBM, while genus‐level identification is impressive, distinguishing between species, an important factor for guiding treatment decisions, has not yet been adequately explored. In PCM, the promise of serum‐based, non‐invasive diagnosis is clear, but the absence of prospective clinical studies and limited evaluation of co‐infections or underlying conditions raises questions about how well these methods will perform in routine healthcare settings.

Moreover, there is a clear lack of standardised protocols for how samples are handled, how spectra are acquired and how data are analysed, making it difficult to compare results across different studies or to apply them consistently in practice. Altogether, while the initial results are promising, larger studies with more diverse patient populations, along with greater methodological standardisation, are essential to confirm the real clinical value of IR spectroscopy. Addressing these gaps will be key to turning this emerging technology into a reliable diagnostic option, especially in low‐resource settings where fast, affordable and accurate fungal diagnosis is most urgently needed.

## 4 Future Directions for IR‐Based Spectroscopy in Medical Mycology

This review brings together the expanding body of evidence on the use of IR spectroscopy for identifying human pathogenic fungi, highlighting how this technology is gradually transitioning from experimental applications to more practical clinical use. The studies discussed here consistently demonstrate that IR‐based approaches, especially when combined with advanced multivariate statistical methods and machine learning, offer a flexible and accurate alternative for fungal identification. Their potential extends beyond traditional research environments, with promising applications in routine clinical laboratories. Much of this progress has been enabled by recent advances in computational tools, which are making these analyses faster, more precise, and accessible to a broader range of laboratories.

However, despite these encouraging developments, important challenges remain before IR spectroscopy can be fully integrated into clinical mycology. One of the most pressing issues is the lack of standardised protocols. There is a clear need for harmonised procedures covering sample preparation, spectral acquisition and data analysis, ensuring reproducibility and consistency across different laboratories. Equally crucial is the development of robust, diverse, and openly accessible spectral databases, especially considering the growing diversity of fungal pathogens and the emergence of region‐specific species.

Among the commercially available tools, the IR Biotyper has emerged as a promising system, bringing IR spectroscopy into a more user‐friendly and clinically applicable format. When compared to MALDI‐TOF MS, which remains the standard for routine microbial identification, the IR Biotyper offers some important advantages. It requires minimal sample preparation, simply transferring a colony directly onto a silicon crystal and eliminates the need for costly reagents, resulting in lower operational costs. Furthermore, the system goes beyond basic species identification, demonstrating the ability to cluster isolates and even detect resistance‐associated spectral patterns, which are aspects where MALDI‐TOF MS still faces limitations.

Nonetheless, it is important to approach these findings with a balanced perspective. The IR Biotyper is still relatively new in the field of clinical microbiology, and its spectral databases, particularly for fungal pathogens, are in continuous development. Although the ongoing operational costs are low, the initial investment in equipment can be a significant barrier for some laboratories. Additionally, MALDI‐TOF MS has benefited from over a decade of optimization and clinical integration, meaning IR‐based systems have some ground to cover in terms of widespread acceptance and validation.

Looking ahead, the future of IR spectroscopy in mycology appears highly promising. Beyond identifying cultured isolates, IR technology has the potential to be adapted for point‐of‐care devices, especially valuable in low‐resource settings where fungal diseases are most prevalent and diagnostic infrastructure is limited. Moreover, IR spectroscopy could play a key role in rapid antifungal resistance detection, providing clinicians with early therapeutic guidance before conventional susceptibility results are available. Another exciting prospect is the direct identification of fungi from clinical samples, such as blood or urine, bypassing lengthy culture steps and potentially shortening time to diagnosis. When combined with complementary tools like genomics or proteomics, IR‐based methods could form the basis of faster, more comprehensive diagnostic workflows.

In conclusion, infrared spectroscopy, and in particular platforms like the IR Biotyper, stands at a crucial point of development. With further improvements in database expansion, standardisation of protocols, and real‐world clinical validation, this technology holds the potential to significantly enhance the diagnosis of fungal infections. While it may not completely replace methods such as MALDI‐TOF MS, it offers complementary advantages, particularly in contexts where affordability, speed and ease of use are essential. If these current limitations are successfully addressed, IR spectroscopy could help bridge existing diagnostic gaps, providing quicker and more accessible solutions to clinicians and ultimately benefiting patient care in fungal disease management.

## Funding

This study was supported by the Fundação Norte‐Rio‐Grandense de Pesquisa e Cultura (FUNPEC; 06/2023), the Conselho Nacional de Desenvolvimento Científico e Tecnológico (CNPq) and the Ministry of Health (Grant 444501/2023‐1), the National Institute of Science and Technology (INCT) Funvir, Brazil (Grant 405934/2022), and the Coordenação de Aperfeiçoamento de Pessoal de Nível Superior (CAPES), Brazil (Finance Code 001). R.W.B. and D.A.S. are research fellows of CNPq (Grants: 309210/2025‐9 and 303762/2020‐9).

## Conflicts of Interest

The authors declare no conflicts of interest.

## Data Availability

The data that support the findings of this study are available on request from the corresponding author. The data are not publicly available due to privacy or ethical restrictions.
